# A Simultaneous Genetic Screen for Zygotic and Sterile Mutants in a Hermaphroditic Vertebrate (*Kryptolebias marmoratus*)

**DOI:** 10.1534/g3.115.022475

**Published:** 2016-01-20

**Authors:** Sofia Sucar, Ginger L. Moore, Melissa E. Ard, Brian C. Ring

**Affiliations:** * Department of Biology, Valdosta State University, Georgia 31698; † Agrobiotechnology Laboratory, National Institute for Agricultural Technology (INTA), Balcarce 7620, Buenos Aires, Argentina; ‡ Department of Neurosurgery, University of Florida, McKnight Brain Institute, Gainesville, Florida 32610; § Whitney Laboratory for Marine Bioscience, Department of Biology, St Augustine, Florida 32080

**Keywords:** *Kryptolebias marmoratus*, forward genetic screen, ENU mutagenesis, zygotic mutant, sterile mutant

## Abstract

The mangrove killifish, *Kryptolebias marmoratus*, is unique among vertebrates due to its self-fertilizing mode of reproduction involving an ovotestis. As a result, it constitutes a simplistic and desirable vertebrate model for developmental genetics as it is easily maintained, reaches sexual maturity in about 100 days, and provides a manageable number of relatively clear embryos. After the establishment and characterization of an initial mutagenesis pilot screen using N-ethyl-N-nitrosourea, a three-generation genetic screen was performed to confirm zygotic mutant allele heritability and simultaneously score for homozygous recessive mutant sterile F_2_ fish. From a total of 307 F_2_ fish screened, 10 were found to be 1° males, 16 were sterile, 92 wild-type, and the remaining 189, carriers of zygotic recessive alleles. These carriers produced 25% progeny exhibiting several zygotic phenotypes similar to those previously described in zebrafish and in the aforementioned pilot screen, as expected. Interestingly, new phenotypes such as golden yolk, no trunk, and short tail were observed. The siblings of sterile F_2_ mutants were used to produce an F_3_ generation in order to confirm familial sterility. Out of the 284 F_3_ fish belonging to 10 previously identified sterile families, 12 were found to be 1° males, 69 were wild-type, 83 sterile, and 120 were classified as */+ (either wild-type or carriers) with undefined genotypes. This screen provides proof of principle that *K. marmoratus* is a powerful vertebrate model for developmental genetics and can be used to identify mutations affecting fertility.

*Kryptolebias marmoratus*, hereafter referred to as *K. marmoratus*, is the scientific name used to designate the common mangrove killifish (also referred to as mangrove rivulus), a species of fish living along the eastern coast of North, Central, and South America, extending from Florida to Brazil primarily among shallow intertidal waters in mangroves. More than 90% of this species are known to exist in nature and in the laboratory as self-fertilizing hermaphrodites (Mackiewicz *et al.* 2006b). Primary males are easily discernible due to their bright orange coloration and lack of ocelli on the tail that is present in hermaphrodites. Their percentage in natural populations can vary from <1% in most natural habitats to 20% at Twin Cays, Belize (Mackiewicz *et al.* 2006a).

Hermaphroditism is common among invertebrates, such as the model organism *C. elegans*, but not as common among vertebrates (Weeks *et al.* 2006). Even though in some cases it can result in reproductive anomalies causing infertility, many fish species are asynchronous hermaphrodites. *K. marmoratus* is a synchronous, self-fertilizing vertebrate among members of its related genus (see review by Avise and Tatarenkov 2015). For this organism, self-fertilization is the natural mode of reproduction resulting in the production of viable progeny through internal fertilization. Juveniles primarily contain ovaries but the majority transition to have an ovotestis (a mixed gonad) configuration after 100 days (Cole and Noakes 1997). *K. marmoratus* exploits a mixed-mating strategy termed androdiecy, mainly reproducing by self-fertilization but occasionally mating with males by external sexual reproduction. For example, Mackiewicz *et al.* (2006a) demonstrated successful laboratory outcrossing of hermaphrodites to males through microsatellite analysis of resulting hybrids. Androdiecy becomes of especial importance, promoting outcrossing for a species living in a highly variable environment, since these challenging conditions may act as selective pressures in nature (Mackiewicz *et al.* 2006c).

Genetic screens have been performed to search for zygotic mutations in various model organisms. Zygotic mutations occur in genes expressed in the embryo after fertilization as maternal transcripts decline, thus being required for continued embryogenesis. Reverse genetic techniques have proven to be useful in the search for zygotic mutations, but require knowledge of the gene or genes under study. Conversely, forward genetic screens do not require preknowledge and are significant because they enable the determination of gene function through phenotypic screening of desired mutant phenotypes applicable to deciphering developmental genetic pathways.

During the 1990s, N-ethyl-N-nitrosourea (ENU; C_3_H_7_N_3_O_2_) mutagenesis was investigated in zebrafish (*Danio rerio*) (Solnica-Krezel *et al.* 1994). This is a highly potent mutagen that induces point mutations in the germ-line that are heritable in the offspring. Several genetic screens were performed in this fish revealing the presence of recessive mutations induced by ENU and their inheritance in the offspring (Mullins *et al.* 1994; Solnica-Krezel *et al.* 1994; Driever *et al.* 1996; Haffter *et al.* 1996). These genetic screens were fundamental because they demonstrated how mutations affecting a variety of developmental processes could be efficiently recovered from zebrafish; a vertebrate model that develops externally with a clear egg chorion. This constitutes a great advantage over other vertebrate models such as mammals in which such mutations are not easily detected due to *in utero* development.

Even though the first studies on the basic ecology and biology of *K. marmoratus* were described more than 50 yr ago (Harrington and Rivas 1958; Harrington 1961), no large-scale genetic screens utilizing its simple self-fertilizing reproduction have been reported. Unlike *K. marmoratus*, zebrafish is a gonochoristic species making genetic screens more laborious because of the time and effort devoted to husbandry and crossing, as well as requiring an extra generation for the identification of homozygous mutants (Pelegri and Mullins 2004). *K. marmoratus* has the potential advantage of making the identification of mutations easier, as a result of genomic isogeny and the ease of self-reproduction. Recently, 21 clonal-isogenic laboratory stocks were established within the scientific community through microsatellite and mitochondrial DNA analysis (Tatarenkov *et al.* 2010). This study further refined the stocks used in the first ENU pilot mutagenesis screen in *K. marmoratus* (Moore *et al.* 2012). Other advantages of this fish for genetic screens include low maintenance husbandry and the versatility of being reared as a colony or as individual specimens for self-crossing, as well as displaying early stage embryonic development through a translucent chorion. Furthermore, a typical well-fed fish, in the range of 20–40 mm in length at its peak reproductive capacity can produce 15–25 eggs per day (Moore *et al.* 2012).

Moreover, *K marmoratus* is useful as a vertebrate model for sterility screens, given the fact that, as mentioned, it develops externally with a clear egg chorion where egg/embryo features, such as viability, can be easily observed. The identification of sterile mutants is of particular importance due to the application of such results in the reproductive biology of higher vertebrates. Recently, it was estimated that 1:5000 human births results in diseases of sexual development (DSD) leading to sterility or intersex conditions in Western cultures and even more frequently in smaller consanguine cultures, suggesting genetic factors play a significant role in DSD (Bashamboo and McElreavey 2014). A sterile mutant in *K. marmoratus* is defined by a characteristic type of oviposition laying nonviable eggs, infertile eggs (no sperm), or no eggs at all. Nonviability of embryos produced by a sterile parent is potentially the result of either a paternal effect where sperm are defective or a maternal effect mutation both halting embryonic development prior to zygotic genes being expressed. The latter are mutations in maternally encoded gene products present in the egg cytoplasm (Abrams and Mullins 2009). Maternal factors control development before the activation of the embryonic or zygotic genome and are required for survival during early development. Infertile eggs are often the result of a paternal effect mutation preventing fertilization leading to an embryo void of the characteristic vitelline membrane space or “halo”. Lastly, in the case of *K. marmoratus* mutagenesis, absence of egg production can potentially be caused by a defect in the ovotestis of the fish. Several scenarios can give rise to this phenotype, from anatomical problems preventing oviposition of developing embryos, to internal gonadal defects causing eggs or embryos to be arrested in a certain stage of development and subsequently deteriorate as described by such studies in zebrafish (Bauer and Goetz 2001). Sterile fish producing nonviable eggs having a maternal effect mutation were described in zebrafish (Dosch *et al.* 2004; Wagner *et al.* 2004). Mutations affecting testes, as well as ovarian mutations, were also discovered in zebrafish (Bauer and Goetz 2001). Lastly, nonegg-laying, sterile fish were described in medaka by Morinaga *et al.* 2004.

This study aims to demonstrate that *K. marmoratus* is a useful vertebrate model for forward genetic screens that can be designed to uncover zygotic and fertility mutants simultaneously with the simplicity of one less generation and ease of self-crossing within hermaphrodites. Studies on gonadal defects making use of this fish constitute a starting point to expand our knowledge on abnormalities causing gonadal malfunction in higher vertebrates. We provide proof of principle that ENU can be used as a mutagen in the identification and heritable confirmation of both zygotic and sterile mutations recovered in an F_3_ generation screen of *K. marmoratus*. Based on data presented here, it is anticipated that this organism will become an advantageous model for genetic studies of multiple fields of comparative biology in the near future.

## Materials and Methods

Every experiment described in this study was approved by the Valdosta State University (VSU) Institutional Animal Care and Use Committee (Animal Use Protocol 00045-2012) under the National Institutes of Health’s Office of Laboratory Animal Welfare Assurance Number A4578-01.

### Genetic screen

*K. marmoratus* were maintained in the VSU Aquatic Laboratory in a controlled environment at 27°−30° on a 14 hr light: 10 hr dark photoperiod. For a detailed description of fish husbandry refer to Supporting Information, File S1.

The genetic screen described here is a continuation of a pilot screen where 34 fish from a parental generation were mutagenized using ENU (Sigma, St. Louis, MO) (Moore *et al.* 2012). Briefly, from 284 fish screened in the first generation (F_1_), 73 were identified that produced zygotic mutants in their F_2_ progeny. On a subsequent rescreen, 71 out of these 73 F_1_ fish reproduced zygotic mutations in the F_2_ progeny in predicted Mendelian ratios. For details on how ENU mutagenesis was performed, refer to Moore *et al.* 2012. We continued our analysis of 47 F_1_ clonal lineages representing a diverse set of zygotic phenotypes from the original 71 confirmed above (66%).

This study focuses on a simultaneous screen to: (a) confirm that the above chosen zygotic mutants are heritable into the next generation (F_3_), (b) simultaneously identify sterile F_2_ fish, and (c) confirm sterility in the F_3_ generation by raising nonsterile sibling fish (*i.e.*, heterozygous carriers) to sexual maturity and scoring their F_4_ offspring.

### Confirmation of zygotic mutants

**Figure 1:**
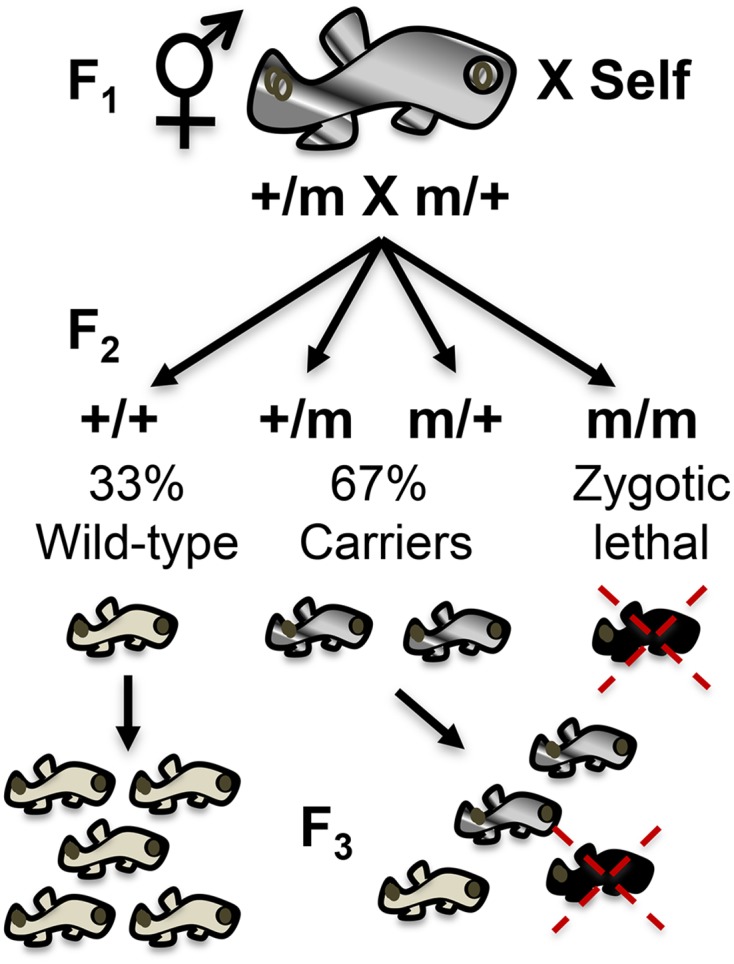
Summary of zygotic confirmation scheme. F_1_ fish previously identified as heterozygous for a zygotic mutation are allowed to self-cross. In their F_2_, they are expected to produce 25% zygotic mutant embryos, which were originally identified as embryonic lethal. Therefore, surviving F_2_ fish are composed of two carriers (*m/+*): one wild-type (*+/+*) ratio with the remaining fish displaying a zygotic defect leading to lethality. F_2_ fish are allowed to self-cross to confirm the zygotic phenotype into the F_3_ and assess proper transmission of wild-type phenotypes.

Mutations producing zygotic defects were originally identified as embryonic lethal. Therefore, surviving F_2_ fish are composed of a 2 carriers (*m/+*)*:* 1 wild-type (*+/+*) ratio with the remaining fish displaying a zygotic defect leading to lethality (Figure 1). For each F_2_ family, at least eight remaining fish were raised to sexual maturity to ensure both *m/+* and *+/+* genotypes were represented from all 47 founding F_1_ fish and used to confirm the zygotic mutant pattern of inheritance into the F_3_. Zygotic mutations manifest as obvious phenotypes during embryogenesis and were therefore relatively easy to identify under the dissecting microscope and to compare to previous observations across generations. Defects in head morphology, eyes and/or tail structure, patterning, pigmentation, *etc*., most being described in other fish species like medaka or zebrafish, were confirmed (Moore *et al.* 2012).

A minimum of 20 F_3_ embryos were collected from each F_2_ fish once or twice a week in petri plates to form an F_3_ clutch, with both the collection date and total number of embryos recorded, including viable and nonviable embryos. All nonviable embryos were removed and the remaining viable ones were observed 2 d later. Embryos were observed for zygotic mutations, categorized according to phenotype, and counted. After scoring a minimum of 20 embryos, the zygotic mutation was confirmed if multiple F_2_ carrier parents (*m/+*) produced F_3_ embryos displaying the same or new phenotypes as the prior generation. The observed and expected values were compared using a chi-square goodness-of-fit test across the F_2_ families.

### Simultaneous identification of sterile fish

F_2_ fish were simultaneously screened for sterility as these types of mutations were predicted to segregate among the mutagenized genomes (*i.e.*, 47 F_1_ families) where the zygotic mutations were previously identified from heterozygous carriers (*m/+*). However, unlike an early zygotic lethal mutant, an F_2_ fish must be raised to adulthood to determine if it is sterile. A fish homozygous for a sterile mutation was predicted to fall into three different phenotypic classes belonging to two types. The first type produces eggs that are nonviable due to maternal effects or unfertilized within a selfing hermaphrodite (Type I). The second type is defective in gonadogenesis or ovotestis development and does not lay eggs (Type II). After collecting a minimum of 20 F_3_ embryos, an F_2_ adult fish was considered sterile if (a) 90% or more of the progeny were nonviable or nonfertilized (Type I) or (b) no progeny were observed for a period greater than 2 months after reaching sexual maturity (∼6 months old; Type II).

### Confirmation of sterile fish

**Figure 2:**
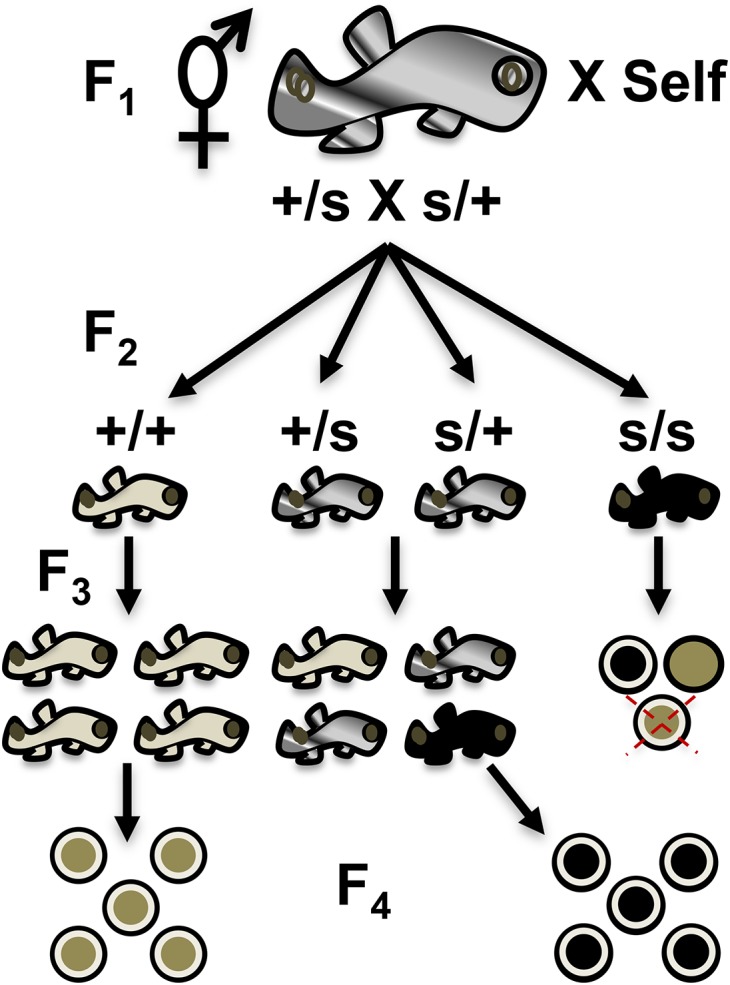
Sterile mutant identification and confirmation scheme. A fish from the F_2_ generation is identified as sterile (*s/s*) when it is raised to adulthood and has no viable progeny belonging to any of two predicted types (see text for details). Among the types are three categories of predicted embryos: (1) black circle with white halo (fertilized but nonviable); (2) tan circle with no white halo (nonfertilized); and (3) tan circle with white halo and red X (no progeny laid). The sterile F_2_ siblings are composed of two carriers (*+/s*): one wild-type (*+/+*). These siblings are self-crossed to collect F_3_ progeny that are in turn raised to sexual maturity. Wild-type F_3_ siblings (*+/+*) will lay 100% wild-type F_4_ viable embryos (tan circle with white halo). F_2_ fish carrying the sterile allele (*+/s*) will give rise to 25% F_3_ sterile progeny that, when raised to sexual maturity, will lay 100% defective embryos or be devoid of F_4_ progeny. Example given is a nonviable maternal effect mutant that arrests during early cleavage (all black circles with halo, see Figure 7).

In the case that an F_2_ fish was found to be sterile, it and its siblings were kept for further analysis. Since the mutation causing sterility makes it impossible to raise progeny from the sterile fish, their siblings were analyzed to confirm sterility into the next generation. For this purpose, two to eight siblings of a sterile F_2_ were allowed to self-cross to produce an F_3_ family (Figure 2). These F_3_ fish were raised to maturity and screened for sterility in separate breeder tanks. Their embryos were collected following the aforementioned procedure. Embryos were collected three times a week and classified as viable or nonviable. Upon initial collection, all nonviable embryos were discarded, while the remaining viable embryos were observed 2 d later and scored as viable or nonviable. If characterization was difficult, embryos were observed under a dissecting microscope to assess viability. Once a fish was categorized as sterile (Type I or II), additional embryos were subsequently collected to a total of n = 100 to further confirm the result and assess penetrance. If a fish was found that did not meet the sterility criteria during this period, it was categorized as *+/+* or *s/+* depending on its siblings’ phenotypes. Existing mutant lines generated in this study are made available upon request.

### Photography of embryos and fish

Unlike the zebrafish chorion, *K. marmoratus*’ chorion is rather difficult to dissect away from developing embryos. Even though in some cases chorions were removed from embryos by the hatching-enzyme method adapted from medaka (Mourabit *et al.* 2011), most digital images in this study correspond to embryos with intact chorions. Visual observations were recorded using an Olympus DP72 camera mounted to an Olympus SZX16 stereomicroscope (Olympus, Center Valley, PA).

### Statistics

Sigma Plot (Systat Software, Inc., San Jose, CA) was used to run a Mendelian chi-square analysis for the confirmation of zygotic mutants, simultaneous identification of sterile fish, and confirmation of sterile fish.

### Data availability

The authors state that all data necessary for confirming the conclusions presented in the article are represented fully within the article.

## Results

From the pilot forward genetic screen in *K. marmoratus* (Moore *et al.* 2012), 73 out of 284 F_1_ fish produced 25% progeny showing zygotic defects in a recessive pattern. Zygotic mutants were classified into eight main phenotypic categories (see Moore *et al.* 2012 for classification details). Subsequently, these 73 F_1_ fish producing zygotic mutants were rescreened by scoring larger numbers of F_2_ offspring to further confirm the initial observations. Seventy-one F_1_ fish (97%) confirmed the previous recessive zygotic mutant phenotype in their F_2_ offspring, representing a 25% hit rate for zygotic lethals from the initial 284 F_1_ genomes screened.

A continuing genetic screen into the next generation (F_3_) is described in order to: (a) confirm zygotic mutant allele heritability (Figure 1) and (b) simultaneously score for homozygous recessive mutant sterile F_2_ fish (*s/s*) (Figure 2). Furthermore, the sterile mutants were confirmed into the F_3_ generation to prove the heritability of the mutations by scoring their siblings’ F_4_ offspring – a form of backward-genotyping.

### Simultaneous screen

From a total of 307 F_2_ fish screened, 10 were found to be 1° males while 16 were sterile (Table 1). From the remaining 281 F_2_ fish, 92 were found to be wild-type and 189 were carriers of zygotic recessive alleles (*m/+*). As part of this simultaneous screen, two to 13 fish were screened per family. Primary males were found ranging from none in some families up to a maximum of two in others, making up 3.6% of the total number of fish screened. This is consistent with natural observations of males (either primary or secondary) found in nature (Laughlin *et al.* 1995). Sixteen sterile mutant hermaphrodites showing maternal effects (nonviable embryos, NV) or ovotestis defects leading to sterility (*s/s*) were identified. On average 1.33 sterile fish were observed per family.

**Table 1:** Summary of simultaneous zygotic and sterile F_2_ screen in *K. marmoratus*

F_2_ Fish	(*+/+*)	(*m/+*)*^a^*	(*s/s*)*^b^*	1° Males	N*^c^*	Genomes Screened
Sum	92	189	16	10	307	38–76
Mean	2	4	1.33	0.21	6.5	–
Range	0–6	0–9	1–3	0–2	2–13	–

Updated from Moore *et al.* (2012).

a Confirmed carriers of zygotic recessive alleles (*m*).

b Sterile mutant hermaphrodites: maternal effects or ovotestis defects (*s/s*).

c N = total F_2_ fish scored.

### Confirmation of zygotic mutants

**Figure 3:**
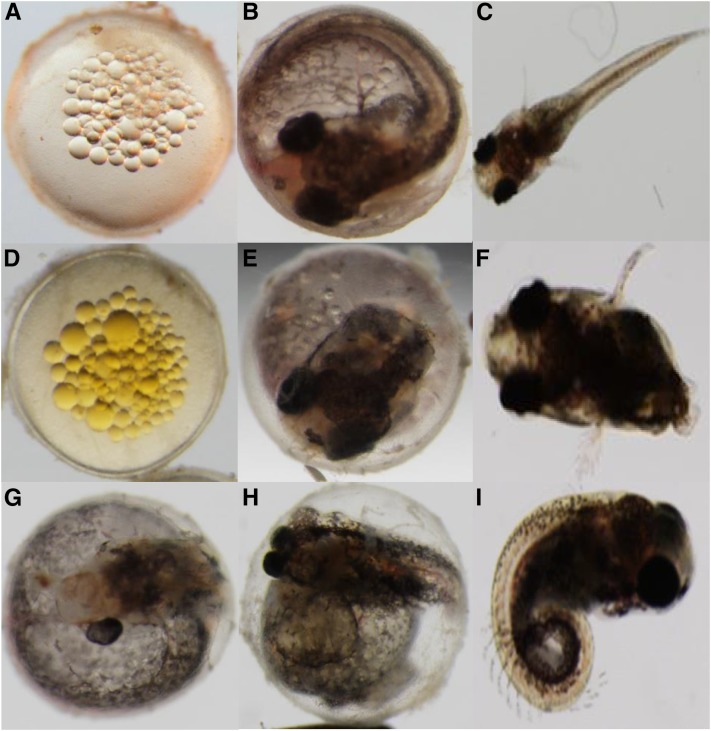
New zygotic phenotypes identified across different developmental stages in the confirmation screen. (A) Wild-type fertilized embryo. (B) Wild-type embryo (14 dpf). (C) Wild-type hatched juvenile fish. **(**D) Golden yolk phenotype (R058 family). (E) No trunk phenotype (R109 family). (F) Short tail phenotype (R228 family). (G) One eye phenotype (R182). (H) Dwarf/eyes forward phenotype (R196 family). (I) Downward curled tail phenotype (R217 family).

As mentioned, from the remaining 281 F_2_ fish, 92 were wild-type (33%) and 189 carriers of the zygotic recessive allele (67%) as expected for the normal recessive mode of zygotic lethal inheritance (Figure 1). Fish were identified as carriers of a zygotic mutation because multiple carriers produced F_3_ progeny showing a zygotic defect found in the previous generation. Most of the zygotic phenotypes discovered in the pilot screen (Moore *et al.* 2012) were confirmed in the present study. Also, as part of this zygotic confirmation, the zygotic mutant categories were expanded because some new phenotypes such as golden yolk, no trunk, and short tail were consistently observed (Figure 3). Finally, mutations classified as “unresolved gastrulation defects” that showed consistent Mendelian patterns of inheritance were observed, but were not further characterized.

The most obvious zygotic defects were those affecting morphology of the tail. They were either curly, reduced/shortened, or absent altogether. Curly tailed mutants were found where the tail was either curled-down or curled-up, with various degrees of penetrance as compared to wild-type siblings. An example of downward curl was observed in the R217 family (Figure 3I).

**Figure 4:**
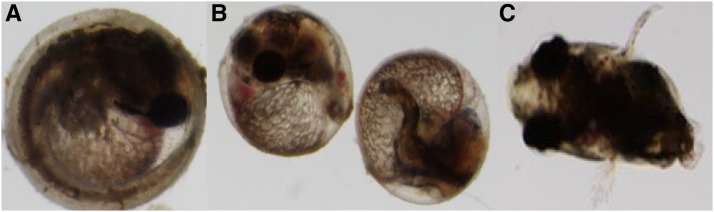
Short tail phenotype across two F_2_ clonal lines of the R228 family (14 dpf). (A) Wild-type embryo. (B) F_3_ embryos descended from R228-6 clonal line (left anterior view, right posterior view). (C) F_3_ embryo descended from R228-8 clonal line.

One of the families screened, R228 (F_1_ founder), consistently gave a short tailed phenotype, with several F_3_ embryos belonging to different F_2_ parents sharing a characteristic reduced/deformed tail fin (Figure 4). The R228-8 (F_2_ descended from F_1_) produced some short tail F_3_ embryos upon hatching. They usually do not survive past this stage and more generally die before hatching. A similar phenotype was discovered in zebrafish resulting from a mutation in a gene called *no tail* (*ntl*; Amacher *et al.* 2002) (refer to the *Discussion* section for more details on this mutation).

**Figure 5:**
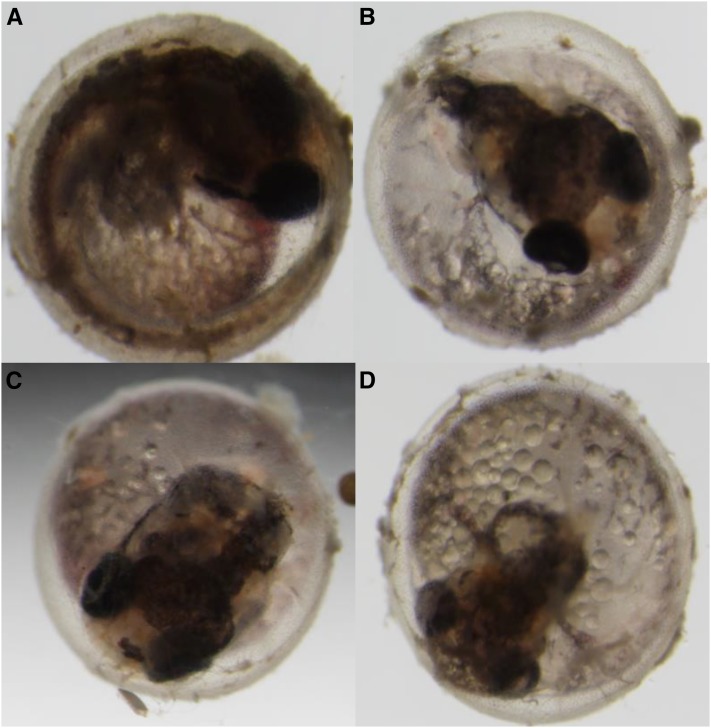
No trunk defect phenotype across multiple F_2_ clonal lines of R109 family (14 dpf). (A) Wild-type embryo. (B) F_3_ embryo descended from R109-3 F_2_ parent. (C) F_3_ embryo descended from R109-4 F_2_ parent. (D) F_3_ embryo descended from R109-8 F_2_ parent.

Among other novel zygotic phenotypes observed, *no trunk*, particularly consisting of essentially a head with no trunk or tail structures, was observed in the R109 family among several F_2_ clonal lineages (Figure 5). This morphology was previously described in medaka as *headfish* by Yokoi *et al.* (2007). The mutant medaka embryo consisted of a fully developed head devoid of trunk and tail and is quite similar to findings presented in this genetic screen (refer to the *Discussion* section for more details on this mutation).

**Figure 6:**
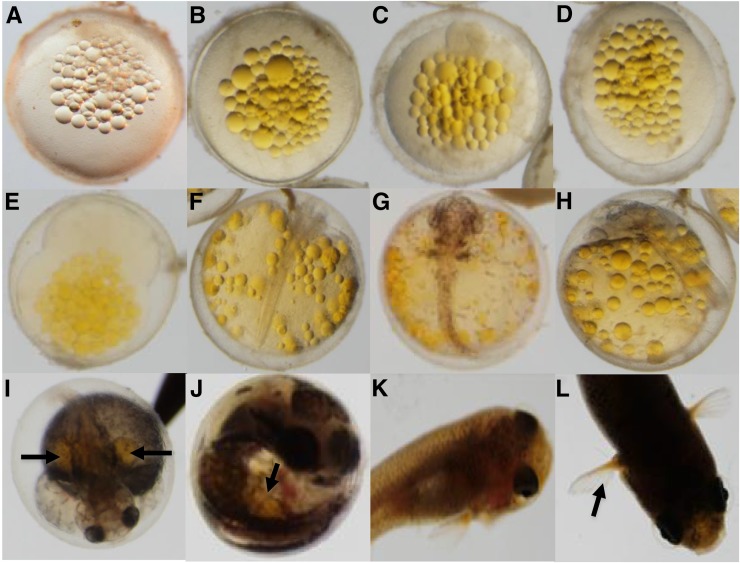
Golden yolk phenotype across development from the R058-2 F_3_ family. (A) Fertilized wild-type embryo with clear oil droplets. B–L. Golden yolk phenotype from embryo to adult among F_4_ offspring. (B) One-cell stage (Stage 1). (C) Eight-cell stage (Stage 4). (D) Early gastrula (Stage 11). (E) Midgastrula (Stage 12). (F) Optic vesicle and somite formation (Stage 17). (G) Dorsal view at liver formation (Stage 26). (H) Side view as pigmentation and body movement increases (Stage 27A). (I) Dorsal view as caudal fin forms (Stage 28). (J) Jaw formation (Stage 30). (K) Juvenile fish. (L) Adult fish with yellow pectoral fin phenotype. Developmental stages are indicated according to Mourabit *et al.* (2011). Arrows indicate location of golden yolk or pigment remnants at later stages of development.

Another new category referred to as *golden yolk*, consistently observed in members of the R058-2 family throughout several developmental stages, was newly identified during the simultaneous screen (Figure 6). This mutation is presumed to be a maternal effect, as the characteristic golden oil droplets are also visible in unfertilized embryos (see Figure S1). The golden phenotype persists throughout development and is visible in the pectoral fins of adult fish (Figure 6L).

We also confirmed our previous observations from the pilot screen (Moore *et al.* 2012). For example, F_3_ embryos with jaw/mouth defects were observed in the R176 and R210 families (see Figure S2). Several families confirmed the eye/skull defect phenotype in their progeny (see Figure S3). Embryos from R234-4 had a characteristic larger eye, while embryos from R182-2 were missing an eye. Eyes were absent in members of the R201 family, which was also characteristic in members of the R152 family, where some of the embryos with no eyes actually survived to the larval stage. F_3_ embryos from the R240 family had forward eyes and skull defects.

Lastly, mutants were found that were categorized as possessing “unresolved gastrulation defects” (see Figure S4). Embryos from the R159 family (Figure S4C) have an unresolved phenotype where no tail or fins are visible and basic structures such as eyes or mouth are not identifiable because there is no evident body plan; the embryo resembles a mass of unorganized cells. Figure S4D shows an F_3_ embryo from the R240 family that in general looks normal, but contains an appendage extending laterally from its side that cannot be classified as any normal structure. Figure S4B shows an image from the R149 family, where the embryo appears thinner than normal, without fully developed eyes and some pigmentation pattern defects. Finally, the mouth/jaw does not appear to be completely developed.

Zygotic confirmation screening also revealed fish from several families exhibiting homozygous viable alleles of a characteristic dark pigment defined as “hyper-pigmented” (see Figure S5). These fish develop normally and grow to adulthood without any further complications. However, in two of these families, R096 and R152, fish are eyeless and possess a typical V-shaped jaw (see arrow in Figure S5, C and D). Their body structure is distorted and slightly twisted. The eye defects in these fish follow Mendelian patterns of inheritance and may explain their hyper-pigmented phenotype due to an environmental response of being blind. In another family, R058, embryos show the hyper-pigmented phenotype but have normal eyes. In such a case, this represents a true breeding clonal line void of other alleles indirectly affecting this phenotype. Interestingly, from this family the clonal line of *golden yolk* phenotype was derived that may result from a carotene deficiency. Within this family, progeny from R058-7 were raised and hyper-pigmentation was observed in embryos belonging to the F_4_ generation (data not shown).

In summary, all but two of the 47 F_1_ families reproduced the zygotic phenotypes or produced new phenotypes upon expansion into the F_2_ generation (Table 2). The lost alleles were associated with small numbers of F_2_ members scored (*e.g.*, R001 only 2 F_2_ fish scored). On average, we found 4/6 heterozygous F2 fish (m/+) per family, consistent with the expected 67% (see Figure 1). The percent of mutants (*m/m*) detected ranged within families from 5 to 69%. However, the average was 23%, consistent with the aggregate total across all F_2_ fish scored and equally important, the mutant phenotypes were consistently observed across multiple F2 clonal lineages.

**Table 2:** Summary of *K. marmoratus* recessive mutants

Family	Category	Phenotype	F_2_*^a^* (%F_3_)*^b^*
R001	Zygotic not confirmed	Wild-type	0/2 (NA)
R002	Sterile I/zygotic	Maternal effect/dwarf	5/6 (14%)
R007	Zygotic	Embryonic lethal	2/8 (13%)
R010	Sterile I and II/zygotic not confirmed	Maternal effect and nonegg layer/wild-type	0/3 (NA)
R013	Zygotic	Dwarf	2/6 (18%)
R015	Sterile I and II/zygotic	Maternal effect and nonegg layer/dwarf and curly tail	4/9 (40%)
R019	Sterile I	Maternal effect and nonegg layer	NA
R058	Zygotic	Golden yolk, dwarf, eye defects, and hyper-pigmentation	6/8 (12%)
R062	Zygotic	Dwarf	5/8 (10%)
R063	Zygotic	Dwarf and skull defects	6/7 (40%)
R064	Zygotic	Embryonic lethal	6/9 (12%)
R066	Zygotic	Embryonic lethal	4/8 (28%)
R074	Zygotic	Eye defects	3/6 (5%)
R075	Zygotic	Eye defects and skull defects	2/6 (24%)
R076	Zygotic	Curly tail	4/6 (16%)
R077	Zygotic	Short tail and jaw defects	4/5 (34%)
R084	Zygotic	Dwarf and jaw defects	4/8 (18%)
R094	Zygotic	Curly tail and skull defects	3/3 (23%)
R096	Zygotic	Dwarf and hyper-pigmentation	4/4 (8%)
R097	Zygotic	Curly tail	3/4 (17%)
R103	Sterile I and II/zygotic	Maternal effect and nonegg layer/curly tail, eye, and skull defects	8/10 (32%)
R107	Zygotic	Embryonic lethal	4/5 (21%)
R109	Zygotic	No trunk	7/8 (33%)
R113	Zygotic	Dwarf, eye, and skull defects	7/8 (13%)
R120	Sterile I/zygotic	Nonegg layer/curly tail and embryonic lethal	4/6 (29%)
R126	Zygotic	Curly tail and embryonic lethal	1/5 (18%)
R129	Zygotic	Curly tail	2/3 (22%)
R130	Zygotic	Dwarf and embryonic lethal	2/3 (11%)
R134	Zygotic	Eye defects and embryonic lethal	4/4 (14%)
R137	Zygotic	Embryonic lethal and curly tail	3/5 (33%)
R149	Zygotic	Curly tail, eye, and/or skull defects	7/10 (25%)
R152	Sterile I and II/zygotic	Maternal effect and nonegg layer/dwarf and curly, no eyes and hyper-pigmentation	4/7 (38%)
R159	Zygotic	Gastrula defects	4/6 (7%)
R171	Sterile II/zygotic	Embryo holder/pigmentation defects	3/3 (44%)
R176	Sterile I/zygotic	Maternal effect/dwarf and jaw defects	1/3 (28%)
R182	Sterile I and II/zygotic	Maternal effect and nonegg layer/dwarf and curly tail with eye defects	5/6 (25%)
R194	Sterile I and II/zygotic	Maternal effect and embryo holder/curly tail, thin-forward eyes, and pigmentation defects	4/5 (69%)
R201	Zygotic	Curly tail, no eyes, and pigmentation defects	7/8 (27%)
R210	Zygotic	Dwarf, jaw/eye defects	4/7 (16%)
R217	Zygotic	Curly tail	7/8 (32%)
R228	Zygotic	No trunk–no tail	7/10 (18%)
R234	Zygotic	Curly tail, skull/eye defects	7/8 (15%)
R240	Zygotic	Curly tail, gastrula defects	8/8 (20%)
R247	Sterile I/zygotic	Maternal effect/curly tail	1/1 (41%)
R248	Zygotic	Embryonic lethal	1/2 (25%)
R249	Zygotic	Curly tail	4/6 (26%)
R257	Zygotic	Curly tail	4/8 (11%)

a The number of F_2_ fish identified as carriers over total screened shown as (*m/+*)/F_2_ total fish.

b Percent of F_3_ embryos displaying the mutant phenotype as an average across the F_2_ (*m/+*) family members (%).

### Goodness-of-fit analysis of data

After analyzing the zygotic mutants’ data by a Mendelian chi-square test (Table 3), the observed numbers were almost exactly as predicted. From a total of 281 F_2_ fish scored, 94 (one third) were expected to be wild-type and 187 (two thirds) were expected to be carriers of the zygotic recessive allele. Results showed that 92 wild-types and 189 carriers were found. Therefore, the null hypothesis was rejected (χ^2^ = 0.064). Thus, the zygotic confirmation data closely matched Mendelian chi-square expectations.

**Table 3:** Zygotic mutants chi-square test summary table

F_2_ Fish	(*+/+*)	(*m/+*)	Total
Observed	92	189	281
Expected	94	187	281
(Obs-exp)^2^/exp	0.043	0.021	0.064

*P* < 0.05, critical value 3.84, df = 1.

### Simultaneous identification of sterile fish

A total of 9095 F_3_ embryos were scored during the simultaneous screen (Figure 2). These embryos belonged to either wild-type F_2_ fish (*+/+*), carriers of the zygotic recessive allele (*m/+*), or sterile mutant hermaphrodites (*s/s*) (Table 4). Sterile F_2_ fish observed during the simultaneous screen showed either maternal effects or ovotestis defects (see *Materials and Methods* section regarding predicted sterile phenotypic classes). Fish with ovotestis defects typically do not lay embryos because these are arrested in early stages of development and remain in the gonad or never completely develop. In some cases, fish with these defects lay a low number of embryos that eventually die. From the total number of F_3_ embryos scored that belong to *s/s* fish, the majority corresponded to F_2_ parents displaying a maternal effect. On average, 21 F_3_ embryos from each F_2_ sterile fish were collected (Table 4). The total number of embryos collected from all F_2_ sterile fish was 334, with a range from zero to 78 per fish. An average of 30 F_3_ embryos from each F_2_ fish across all genotypes, ranging from zero (Type II, no embryos) to 164 embryos per fish, were scored. Carriers of the zygotic recessive allele laid 6131 F_3_ embryos, while wild-type F_2_ fish laid 2630 as expected since the number of carriers constitutes the larger genotypic class among the families.

**Table 4:** Summary of F_3_ embryos scored in the simultaneous screen

F_3_ Embryos From:	(*+/+*)	(*m/+*)*^a^*	(*s/s*)*^b^*	N*^c^*
Sum	2630	6131	334	9095
Mean	29	32	21	30
Range	3–164	4–89	0–78	0–164

a Confirmed carriers of zygotic recessive alleles (*m*).

b Sterile mutant hermaphrodites: maternal effects or ovotestis defects (*s/s*).

c N = total F_3_ embryos scored.

### Confirmation of sterile fish

In order to confirm sterile mutants, F_3_ sibling fish were raised to sexual maturity. A total of 284 F_3_ fish belonging to 10 previously identified sterile families were screened (Table 5 and Figure 2). This corresponds to 46 F_2_ siblings that were raised to sexual maturity to produce the 284 F_3_ fish screened. An average of 4.6 F_2_ siblings were set up per family, ranging from one to eight F_2_ fish per family. From the 284 F_3_ fish screened, 12 were found to be primary males, with a range from zero to two males per family. From the remaining 272 F_3_ fish, 69 were wild-type (*+/+*), 83 were sterile (*s/s*), and 120 were either wild-type or carriers, referred to as */+ because their genotypes are as yet undefined. In order to determine their genotype, their progeny would need to be raised to maturity and scored for continued transmission of mutant alleles. Therefore, it should be possible to determine in the future if they are either wild-type (*+/+*) or carriers of mutant sterile alleles (*s/+*). For the confirmation into the F_3_, this was not necessary and this is why they are only classified as */+ en masse in this study. Sterile F_3_ fish ranged from one to 10 per family with an average of about two F_3_ sterile fish per family (1.8). Wild-type F_3_ fish ranged from zero to nine depending on the family (average of 4.6 fish), averaging 4.14 fish with a */+ genotype per family, ranging from zero to eight.

**Table 5:** Confirmation of sterile mutants

	F_2_ Families	F_2_ Siblings	F_3_ *+/+*	F_3_ */+	F_3_ *s/s*	1° Males	N*^a^*
Sum	10	46	69	120	83	12	284
Mean	–	4.6	4.6	4.14	1.8	0.26	6
Range	–	1–8	0–9	0–8	1–10	0–2	1–11

a N = total F_3_ scored.

F_4_ embryos collected during the sterile confirmation screen were analyzed for viability (Table 6). A total of 8439 F_4_ embryos were scored, with an average of 31 per F_3_ parent. The range per fish varied from zero to 119. From the total of 8438 F_4_ embryos, 3922 were viable and 4516 were nonviable. As expected from a sterile screen, the number of nonviable embryos was observed to be higher than the number of viable ones.

**Table 6:** Summary of F_4_ embryos scored in the confirmation of sterile mutants

F_4_ Embryos	Viable	Nonviable	N*^a^*
Sum	3922	4516	8438
Mean	14	17	31
Range	0–42	0–112	0–119

a N = total F_4_ embryos scored.

**Figure 7:**
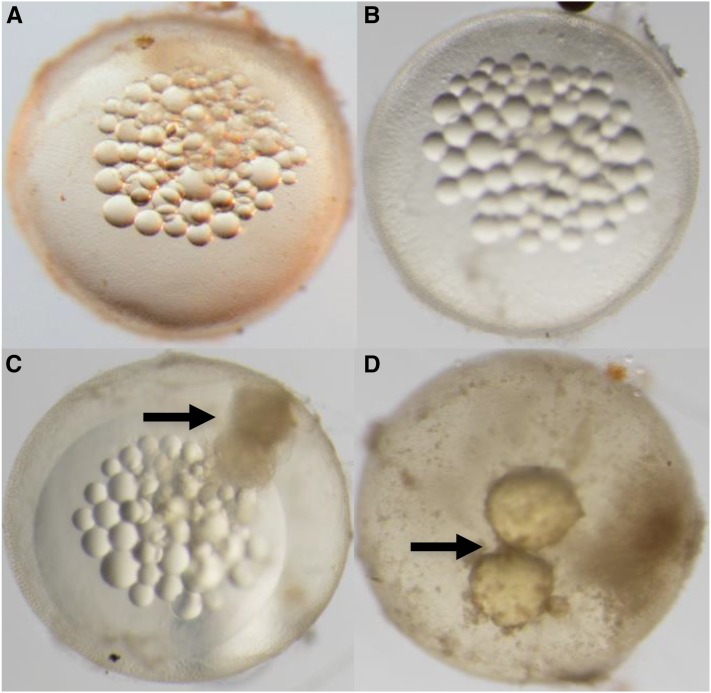
Early embryonic developmental arrest of F_4_ embryos from the confirmation screen (R152-1 family). (A) Wild-type fertilized embryo. (B) Fertilized F_4_ mutant embryo. (C) Within 4 hr development halts at the 2-cell stage and yolk begins to degenerate. (D) Within 24 hr the mutant is completely arrested in development. Arrow indicates 2-cell blastula.

Few of the sterile families analyzed showed either a maternal effect or an ovotestis defect, rather, most of them showed a combination of both (Table 2). Maternal effect sterile mutants are characterized by laying 100% nonviable progeny. Embryos are fertilized, as evidenced by a perivitelline membrane space, but progress to nonviable during early development. For example, F_3_ embryos belonging to the R152 family developed into nonviable embryos by day 2 (Figure 7). They arrest in the 2-cell cleavage stage, the yolk retracts and shrinks, the cell mass degrades, and the embryo finally turns into a degraded chorion characteristic of death.

**Figure 8:**
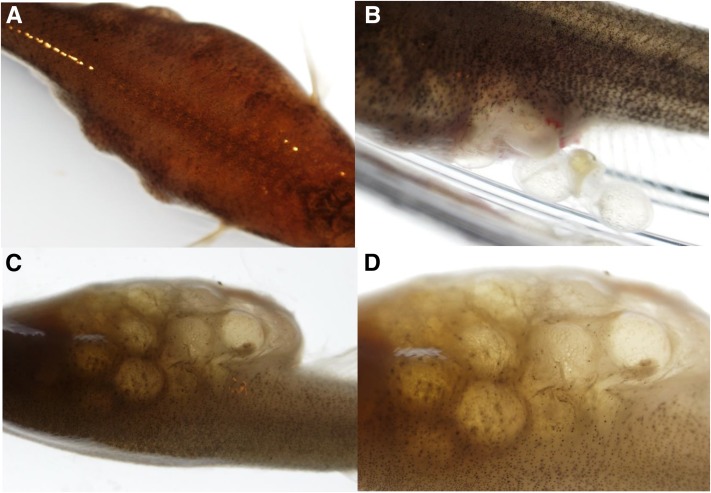
Sterile embryo holder phenotype in F_3_ fish from the R171 family (9 months old). (A) Dorsal view of enlarged abdomen. (B) Side view cloacal damage from inability to lay embryos. (C) Side ventral view of abdomen. (D) Enlarged view of (C) displays embryos in advanced stages of development.

Fish from a category termed embryo holders were also identified (Figure 8). These fish are suspected to possess an anatomical defect causing them to hold embryos within their gonadal lumen upstream of the cloaca. In these cases, embryos are actually formed and fertilized, but the anatomical defect prevents fish from laying them. In fish from the R171 family, the entire abdominal region enlarges as embryos accumulate (Figure 8A). These fish have severe difficulties laying embryos, causing cloacal damage (Figure 8B). Embryos continue to develop to later stages of development after normal oviposition (internal fertilization) but cannot be laid (Figure 8, C and D).

### Goodness-of-fit analysis of data

Sterile siblings’ data were analyzed using a chi-square test (Table 7). From the total number of 46 F_2_ siblings screened, 15 (one third) were expected to show a wild-type phenotype (*+/+*) and 31 (two thirds) were expected to be carriers of the recessive sterile allele. Since the observed number of wild-types was 14 and the observed number of carriers was 32, the null hypothesis was rejected (χ^2^ = 0.099).

**Table 7:** Sterile siblings chi-square test summary

F_2_ Fish	(*+/+*)	(s*/+*)	Total
Observed	14	32	46
Expected	15	31	46
(Obs-exp)^2^/exp	0.067	0.032	0.099

*P* < 0.05, critical value 3.84, df = 1.

### Analysis by families

Table 8 shows results for the 10 families that made it through the confirmation screen. For the R176 and R247 families, the sterile mutation was lost, since no sterile F_3_ fish were found during the confirmation screen, probably due to low numbers of F_3_ fish raised to sexual maturity (Table 8). For five of the families, the percent of F_3_ sterile fish among siblings descended from F_2_ *s/+* parents closely matched the expected 25%. For example, within the R015 line, exactly 25% of F_3_ fish were sterile. For the R010 and R019 families, the percentages of F_3_ sterile fish were 30% and 27.66%, respectively. For R182 and R194 families, the percentages of F_3_ sterile fish were 17.9% and 16.7%, respectively. For three of the families, we observed a number of sterile fish higher than expected. For the R002 and R103 families, 55.6% and 53.6% respectively of the F_3_ fish among siblings descending from F_2_ *s/+* parents were sterile. Finally, 71.4% of sterile progeny were found in the R152 family, which was much higher than the expected percentage for a simple recessive mutation. In some cases, all F_3_ fish from an F_2_ parent were sterile. For example, in two of these lines, R103 and R152, all F_3_ fish from an F_2_ parent were sterile. These three sterile mutant alleles are likely the result of partial dominant maternal effects.

**Table 8:** Summary of sterile mutants across F_2_ families

Sterile Line	F_2_ *+/+*	F_2_ s*/+*	F_2_ Scored	F_3_ Scored	F_3_ (*+/+*)	F_3_ (*/+)	F_3_ s/s	%*^a^*
R002	2	3	5	21	3	8	10	55.5
R010	2	2	4	23	13	7	3	30.0
R015	1	3	4	21	1	15	5	25.0
R019	1	7	8	56	9	34	13	27.6
R103	1	6	7	44	5	18	21	53.5
R152	0	6	6	35	0	10	25	71.4
R176	2	0	2	4	4	0	0	–
R182	1	4	5	34	6	23	5	17.8
R194	3	1	4	30	24	5	1	16.6
R247	1	0	1	4	4	0	0	–

a Expressed as percent of F_3_ sterile fish among siblings descended from F_2_ *s/+* parents.

### Goodness-of-fit analysis of five sterile alleles

Data corresponding to the F_3_ generation of the above five sterile alleles was analyzed using a chi-square test (Table 9). The test focused on F_3_ fish from families R010, R015, R019, R182, and R194 that descend from an F_2_ *s/+* parent. If these families follow Mendelian inheritance, it is expected that 75% of the F_3_ progeny will be *s/+*, *+/+*, or *+/s*. Since these genotypes cannot be distinguished unless they are raised to adulthood (*i.e.*, backward-genotyping), they are symbolized as */+ (see Figure 2). Following the same logic, the rest of the F_3_ fish, which corresponded to 25% of the progeny, were expected to be recessive sterile mutants. From a total of 111 F_3_ fish descending from F_2_ *s/+* parents that were analyzed, 83 were expected to be */+ and 28 *s/s*. Eighty-four */+ and 27 *s/s* fish were obtained. Thus, the null hypothesis is rejected and these five sterile alleles are homozygous recessive (χ^2^ = 0.048).

**Table 9:** Chi-square test of five recessive sterile lines in the F_3_ generation

F_3_ Fish	(*/+)	(s/s)	Total
Observed	84	27	111
Expected	83	28	111
(Obs-exp)^2^/exp	0.012	0.036	0.048

*P* < 0.05, critical value 3.84, df = 1.

### Pedigree analysis

**Figure 9:**
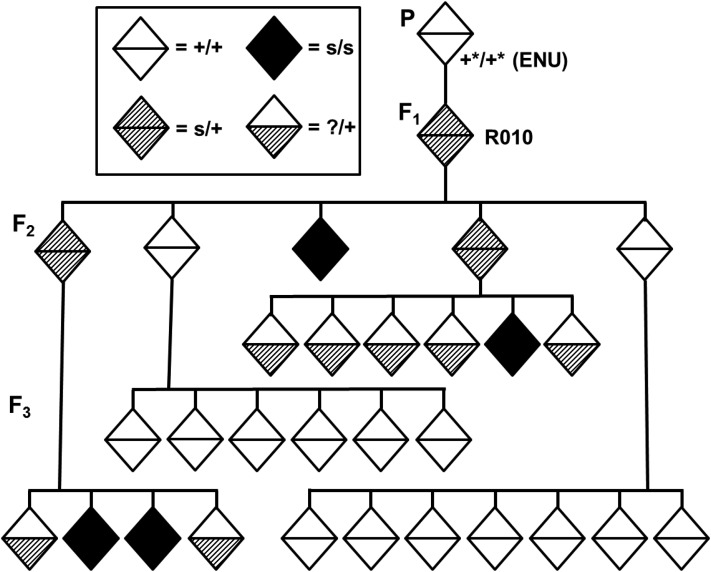
Pedigree of the R010 sterile mutant family displays the heritability pattern of the s allele. Diamonds represent various genotypes of adult hermaphroditic fish within the family (aged >6 months).

It is simpler to observe the overall pattern of inheritance of a sterile allele as displayed in a pedigree. Thus, a pedigree of the R010 sterile mutant family is presented to serve as an example to better visualize inheritance of a mutant allele “*s*” within a family (Figure 9). A single hermaphroditic fish from parental generation P was mutated utilizing ENU and its wild type and heterozygous F_1_ progeny, potentially carrying the sterile allele *s*, were raised to sexual maturity. When these F_1_ fish (*s/+*) self-fertilized, it was predicted that they produced 25% homozygous recessive sterile mutants (*s/s*) in the F_2_ generation if the original *s* allele were segregating in the founder P mutated fish. In the case of the R010 family, F_3_ fish were reared to sexual maturity belonging to four sibling F_2_ fish of the original sterile fish (black diamond). By raising the F_3_ progeny (23 in the R010 family) to sexual maturity and scoring their F_4_ progeny, backward-genotyping is achieved. By this method, two of the original F_2_ siblings were classified as carriers of the sterile allele (s/+) because they founded F_3_ progeny that were either 100% nonviable (Type I maternal effect) or eggless (Type II ovotestis defect). Thus, among the original F_2_ sibling fish, two were wild-type (40%) for the sterile mutation *s* (*+/+*; clear diamond) and two (40%) carriers of the sterile allele *s* (*s/+*; striped diamond). Wild-type F_2_ fish were characterized as such because all of their F_3_ progeny raised to sexual maturity were fully fertile. Conversely, carriers are identified as such because ∼25% (or greater) of their F_3_ progeny are sterile homozygous recessive mutants.

## Discussion

This study serves as proof of principle that *K. marmoratus* is an optimal model organism tractable toward forward genetic screens. Different common phenotypic classes of zygotic mutants were identified in the F_2_ generation and confirmed into the F_3_ generation. Some of these such as tail defects or head structure defects have been previously described in zebrafish (*D. rerio*) (Mullins *et al.* 1994; Solnica-Krezel *et al.* 1994). For example, a family was identified (R228) that consistently gave a short tail phenotype (see Figure 4); a zygotic defect previously identified in zebrafish (Amacher *et al.* 2002). The gene responsible for this phenotype in zebrafish is called *no tail* (*ntl*) and it is hypothesized that a mutant allele of an *ntl* ortholog in *K. marmoratus* is causing this shortened tail phenotype. *Ntl* is an ortholog of the *Brachyury* gene, a member of the T-box family of transcription factors, originally discovered in mice (Dobrovolskaïa-Zavadskaïa 1927). A homozygous *Brachyury* mutation in mice is lethal at around embryonic day 10. Unlike the mouse, zebrafish heterozygous for mutations in the *Brachyury* ortholog of *ntl* do not exhibit any obvious phenotypic defects, which is indicative of a recessive mode of inheritance; they would have to be homozygous for *ntl* mutations to show a phenotype closely resembling the mouse mutants (Showell *et al.* 2004). Zebrafish *ntl* mutant embryos fail to form a differentiated notochord, lack posterior structures, and have abnormally shaped anterior somites. In our putative *ntl K. marmoratus* mutant, we hypothesize that a mutation was introduced into the *ntl* ortholog causing this shortened tail phenotype. Further phenotypic and molecular characterization of the *ntl* mutant alleles is required to confirm this hypothesis.

Another new zygotic phenotype uncovered during the simultaneous confirmation screen was no trunk (see Figure 5), hypothesized to be caused by an ENU-induced mutation in the fibroblast growth factor receptor 1 gene (*fgfr1*), required for proper mesoderm formation. Interestingly, this phenotype has been previously reported in medaka (*Oryzias latipes*), where it received the name of “headfish” and was caused by a point mutation in one of the exons of the fgfr1 signaling factor (Yokoi *et al.* 2007). Studies in zebrafish have analyzed the role of *fgf* during gastrulation (Griffin *et al.* 1995). They reported that the expression of *ntl* is regulated by *fgf*. Inhibition of fgf receptor-signaling leads to the complete loss of the trunk and tail. However the *ntl* mutant lacks the tail and notochord but has an otherwise normal trunk. This demonstrates that trunk development is dependent upon an unidentified gene, or set of genes, referred to as *no trunk* which is regulated by the *fgf* signaling pathway. A future aim is to molecularly characterize the above candidate genes from the short tail and no trunk clonal lineages to determine if ENU-induced mutations are the genotypic cause of the phenotype similarly observed in zebrafish and medaka.

Several viable nonzygotic mutants were identified in this screen such as the hyper-pigmented phenotype. Albeit this could potentially be a true zygotic defect caused by ENU, alternatively, hyper-pigmentation may result as an adaptive response to the environment. Since this phenotype was found in fish with eye defects or absence of the eye, the inability to see might cause visual adaptation leading to hyper-pigmentation as sometimes results in similar zebrafish mutants (Fleisch and Neuhauss 2006). Therefore it is possible that the hyper-pigmentation observed may appear in response to environmental cues such as sight. Indeed, *K. marmoratus* exhibits phenotypic plasticity in other traits in response to environmental conditions (Earley *et al.* 2012). However the nonblind hyper-pigmented mutants uncovered in our screen demonstrate a different class of alleles. In both of these cases, viable progeny could not be collected to determine if the phenotype is also visible in the next generation. In the R096 family, R096-9 laid 100% nonviable embryos. In the R152 family, R152-10 did not lay any embryos. Thus further phenotypic characterization of the hyper-pigmented phenotype was complicated by the intrasegregation of sterile alleles in the F_3_ generation hindering our further propagation toward future characterization of the hyper-pigmented phenotype.

Golden yolk mutants found during this screen were selected to form a true breeding stock void of maternal sterile alleles (see Figure S1). This is the only maternal /zygotic defect that was not lethal and has proven to be true breeding. Currently, members of the F_2_, F_3_, and F_4_ generation, all expressing this unique phenotype, are being raised in the VSU Aquatic Laboratory. A future direction of interest is to analyze this mutation in detail to confirm the maternal effect and to determine if it is the result of a defect in a carotene biochemical synthesis pathway. This novel mutation is of especial importance because it could constitute a useful marker for outcrossing and genetic mapping in *K. marmoratus*, due to its viable zygotic phenotype in later stages of development as well.

It is anticipated that *K. marmoratus* will become a model organism for various genetic screens because of its simplistic advantages as a vertebrate model of development. Compared to gonochoristic species such as zebrafish, it requires one less generation during mutagenesis when utilizing the hermaphroditic parent (Dosch *et al.* 2004). It is easy to maintain and grow in the laboratory and does not require the amount of husbandry of model fish such as zebrafish. *K. marmoratus* are grown in stagnant brackish water and are fed a single source of brine shrimp (*Artemia nauplii*) from hatching to adulthood. Husbandry space is reduced in comparison to similar screens performed in other established models like zebrafish or medaka that require a higher number of F_2_ fish and extensive labor performing crosses between males and females. On the other hand, even though self-fertilization reduces the number of generations needed for genetic screens by one and simplifies animal husbandry, there is a possibility for deleterious mutations to accumulate through self-crossing, which increases lethality via inbreeding and a lack of the ability to perform outcrosses or complementation tests. However, true breeding stocks such as *golden yolk* were selected in this screen, thereby overcoming the deleterious mutation load.

Results from the sterility screen allowed the identification of fish possessing either a maternal effect (Type I; n = 2) or ovotestis defects (Type II; n = 2) or in many cases a combination of both (n = 8). Paternal effect mutants, analogous to those reported in zebrafish with reproductive defects affecting spermatogenesis (Bauer and Goetz 2001), were not found and this constitutes a potential avenue for future investigations. Newsome (2011) originally identified three F_2_ sterile mutants (R002F, R010A, and R019C) out of 35 fish in a preliminary sterility pilot screen. Upon further analysis of these lines, two produced 100% nonviable offspring (R002F and R010A) and one was a nonegg-laying mutant that did not produce any embryos after reaching sexual maturity (R019C). Histological examination of the ovotestis of the R019C fish was indistinguishable from tissue observed in wild-type fish. However, inflammatory cells surrounding oocytes were also observed, and are indicative of an ovarian anomaly. To establish that this inflammation phenotype is indeed a heritable mutant allele, the nonegg layer phenotype was confirmed across F_3_ fish descended from the R019 family during the present screen. Therefore, these confirmatory fish need only be subjected to histological examination to further establish the phenotype from our confirmed genotype.

Among the nonegg-laying mutants identified and confirmed in the extensive screen, gonadal defects are predicted to be seen at the histological level. This is particularly important for nonegg-laying mutant fish because it will be especially interesting to determine what happens inside the mixed gonad leading to sterility. Furthermore, the specific ovotestis mutant phenotype, such as the developmental arrest of oogenesis or major anatomical defects leading to fish unable to ovideposit fully developed embryos, might be identified. In summary, we expect visible defects to be observed in the ovotestis of nonegg-laying sterile fish by histology, in future analyses. Other future directions stemming from this project include the molecular characterization of genes responsible for sterility in *K. marmoratus* identified in this study. Potential candidate genes implicated in diseases of sex determination in humans may be uncovered.

In short, there is much research left to further characterize the sterile families identified in this genetic screen and to identify the stages where nonviable mutant embryos die within each family in order to explore the causes of lethality at the cellular and molecular level. Studies on gonadal defects in *K. marmoratus* would constitute a starting point to expand on the knowledge of abnormalities causing gonadal malfunctioning in humans. If single genes are discovered or if genetic pathways become better understood, this work could potentially be used to advance human reproductive health in diagnosing genetic causes of human sterility. For those who study fish, it would also be important to know more about sterile families showing both maternal effects and ovotestis defects, since these have not been described simultaneously in other fish models. Maternal effects and ovotestis defects could potentially result from the same mutant allele as a result of penetrance effects. Further research is needed to address this hypothesis.

The approach used in the present study has the benefit of allowing the identification of zygotic mutants in the F_2_ generation and the simultaneous confirmation of zygotic recessive mutant alleles and identification of steriles into the F_3_ generation. When the F_2_ generation is grown to adulthood and their F_3_ progeny screened, it is possible to simultaneously confirm zygotic mutants and detect sterile F_2_ fish. It is not necessary to look at F_4_ embryos to identify steriles, only to confirm them. Analogous studies in zebrafish, where natural crosses are performed following ENU treatment, require screening into the F_4_ generation among extended consanguine families where 1/16 of fish among intracrosses are predicted to harbor homozygous sterile alleles (Dosch *et al.* 2004; Wagner *et al.* 2004). Also, in order to avoid interbreeding defects, and since a mapping scheme is involved in these types of zebrafish studies, F_1_ heterozygous fish cannot be crossed to their heterozygous siblings (which would allow for collection of homozygous steriles in the F_2_ generation). F_1_ fish have to be outcrossed with fish from a different strain, but being wild-type for the mutation of interest. This introduces one more generation for the identification of steriles. In an effort to reduce time and space, zebrafish researchers work with extended F_3_ families, which consist of an F_3_ generation resulting from several individual crosses pooled together in the same tank. Not only is space reduced in a *K. marmoratus* screen, but also, the chance of finding a sterile fish is 25% compared to only 6.25% in zebrafish (or any gonochoristic genetic model) through our genetic crossing scheme presented here. Thus, the number of fish necessary to identify homozygous sterile mutants is lower and hence the number of genomes to be screened to reach potential saturation. This is not necessarily related to the type of screen performed, but it is inherently derived from the utilization of the only known self-fertilizing fish. For example, in order to obtain 90% probability levels for identifying maternal effect mutants, 35 individual F_3_ intracrosses are required in zebrafish sterile screens. When *K. marmoratus* are deployed, only eight F_2_ fish need to be set up and allowed to simply self-cross to obtain an equal probability of 90%. Not only is the number of fish needed much smaller but they also belong to a previous generation and do not need to be crossed. Therefore, multiple screens (zygotic and sterile) can be performed in *K. marmoratus* very efficiently. It is anticipated that *K. marmoratus* may serve as a complementary rather than emerging model organism in future genetic studies within the scientific community.

## 

## Supplementary Material

Supporting Information
