# Supplementary material for: A Simultaneous Genetic Screen for Zygotic and Sterile Mutants in a Hermaphroditic Vertebrate (Kryptolebias marmoratus)
Source: G3 (Bethesda). 2016 Jan 20;6(4):1107–19. doi: 10.1534/g3.115.022475 (PMC4825645; doi:10.1534/g3.115.022475)
Supplement: Supporting Information [file supp_6_4_1107__index.html]

A Simultaneous Genetic Screen for Zygotic and Sterile Mutants in a Hermaphroditic Vertebrate (Kryptolebias marmoratus) — Supporting Information 

# A Simultaneous Genetic Screen for Zygotic and Sterile Mutants in a Hermaphroditic Vertebrate (*Kryptolebias marmoratus*)

## Supporting Information for Sucar *et al.*, 2016

**Files in this Data Supplement:**

- Figure S1 - Non-fertilized F3 golden yolk embryos from the R058 family. (.pdf. 85 KB)
- Figure S2 - Jaw/mouth defect mutants (F3 embryos 14 dpf). (.pdf, 91 KB)
- Figure S3 - Eye/skull defect mutants (F3 embryos 14 dpf). (.pdf, 125 KB)
- Figure S4 - Unresolved gastrulation defect mutants (F3 embryos 14 dpf). (.pdf, 100 KB)
- FigureS5 - Hyperpigmentation mutants (mature adult fish). (.pdf, 99 KB)
- File S1 - Fish Husbandry. (.pdf, 81 KB)
