# Supplementary material for: A Simultaneous Genetic Screen for Zygotic and Sterile Mutants in a Hermaphroditic Vertebrate (Kryptolebias marmoratus)
Source: G3 (Bethesda). 2016 Jan 20;6(4):1107–19. doi: 10.1534/g3.115.022475 (PMC4825645; doi:10.1534/g3.115.022475)
Supplement: Supporting Information [file supp_g3.115.022475_FigureS1.pdf]

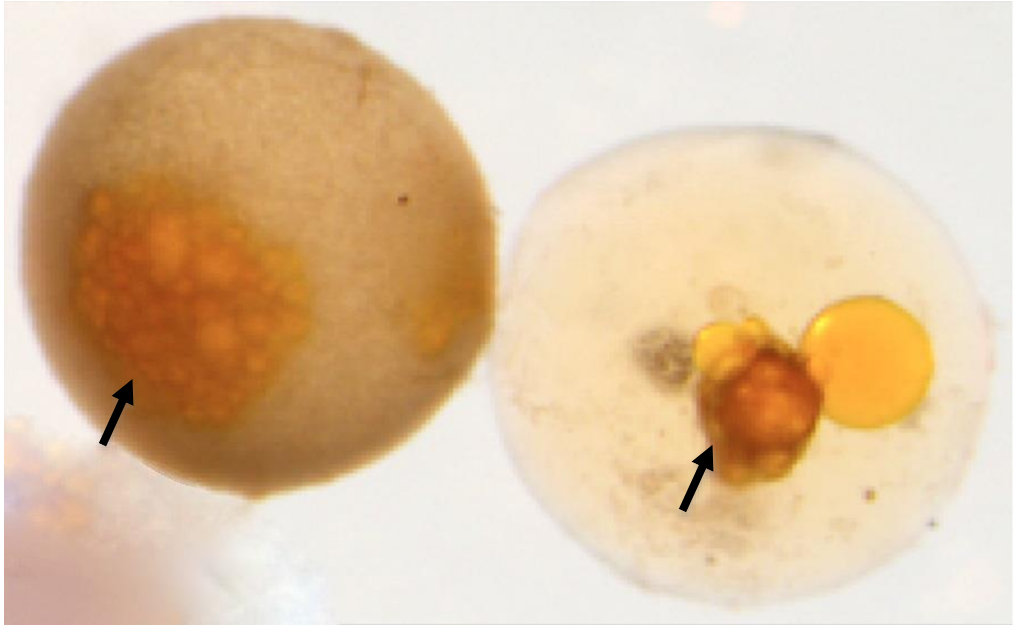

Figure S1. Non-fertilized F_3 golden yolk embryos from the R058 family. The sterile allele segregates with the golden yolk phenotype. Arrows indicate location of golden oil droplets.
