# Supplementary material for: A Simultaneous Genetic Screen for Zygotic and Sterile Mutants in a Hermaphroditic Vertebrate (Kryptolebias marmoratus)
Source: G3 (Bethesda). 2016 Jan 20;6(4):1107–19. doi: 10.1534/g3.115.022475 (PMC4825645; doi:10.1534/g3.115.022475)
Supplement: Supporting Information [file supp_g3.115.022475_FigureS2.pdf]

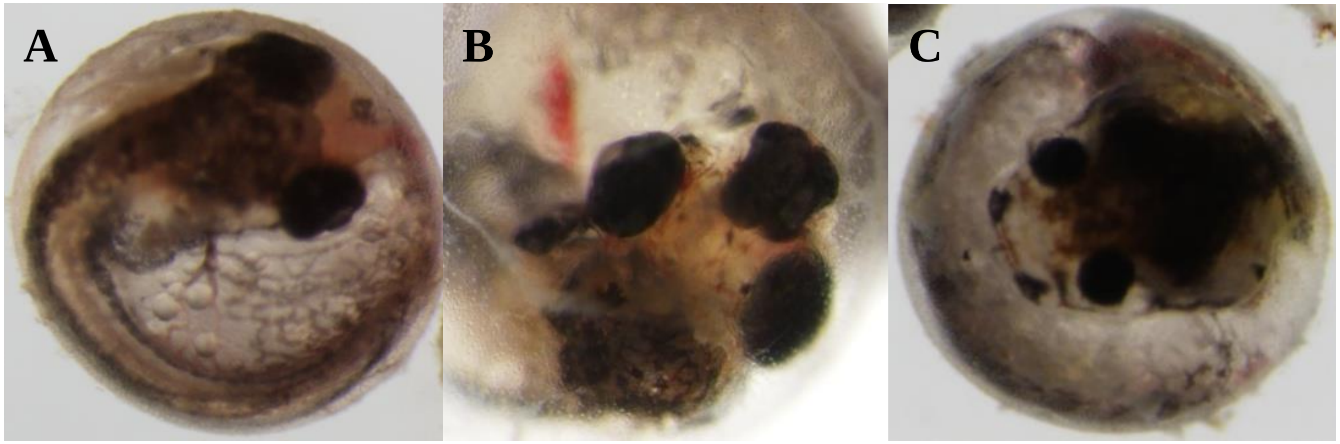

Figure S2. Jaw/mouth defect mutants (F_3 embryos 14 dpf). **A.** Wild type. **B.** Fused jaw/mouth phenotype (R176 family). **C.** Open jaw/eye defect phenotype (R210 family).
