# Supplementary material for: A Simultaneous Genetic Screen for Zygotic and Sterile Mutants in a Hermaphroditic Vertebrate (Kryptolebias marmoratus)
Source: G3 (Bethesda). 2016 Jan 20;6(4):1107–19. doi: 10.1534/g3.115.022475 (PMC4825645; doi:10.1534/g3.115.022475)
Supplement: Supporting Information [file supp_g3.115.022475_FigureS3.pdf]

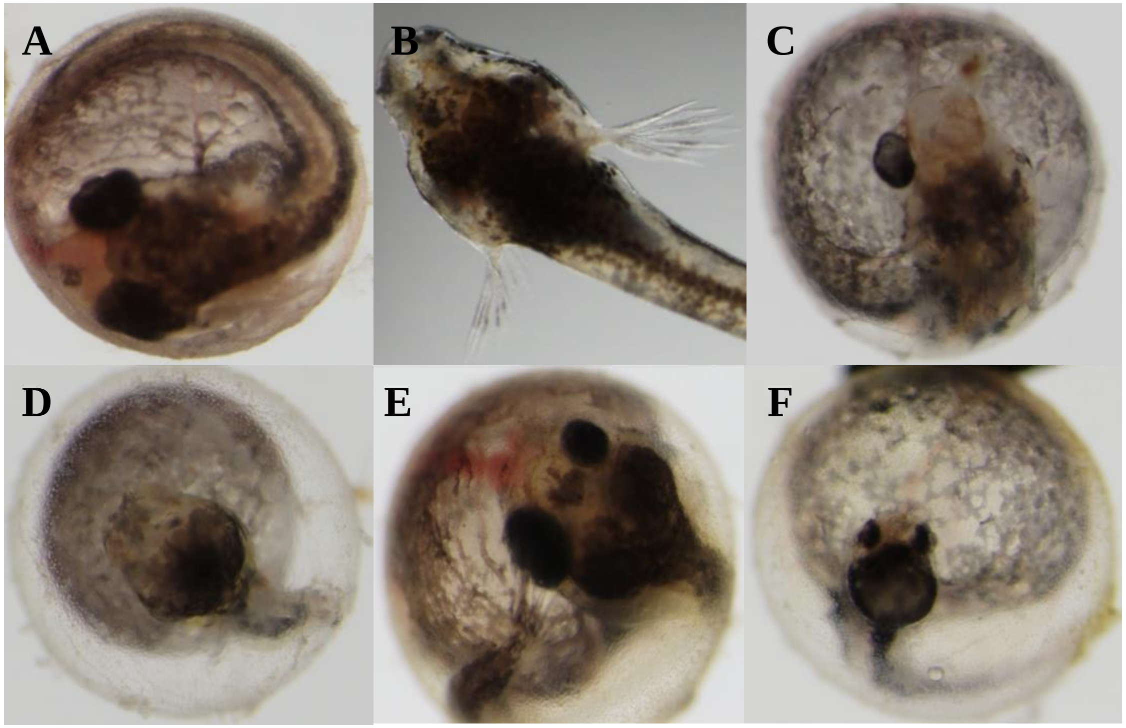

Figure S3. Eye/skull defect mutants (F_3 embryos 14 dpf). **A.** Wild-type. **B.** No eyes phenotype surviving to hatched larval stage (R152 family). **C.** One eye phenotype (R182 family). **D.** No eyes and shortened body axis phenotype (R201 family). **E.** Delayed eye development phenotype (R234 family). **F.** Forward eyes/skull defect phenotype (R240 family).
