# Supplementary material for: A Simultaneous Genetic Screen for Zygotic and Sterile Mutants in a Hermaphroditic Vertebrate (Kryptolebias marmoratus)
Source: G3 (Bethesda). 2016 Jan 20;6(4):1107–19. doi: 10.1534/g3.115.022475 (PMC4825645; doi:10.1534/g3.115.022475)
Supplement: Supporting Information [file supp_g3.115.022475_FigureS4.pdf]

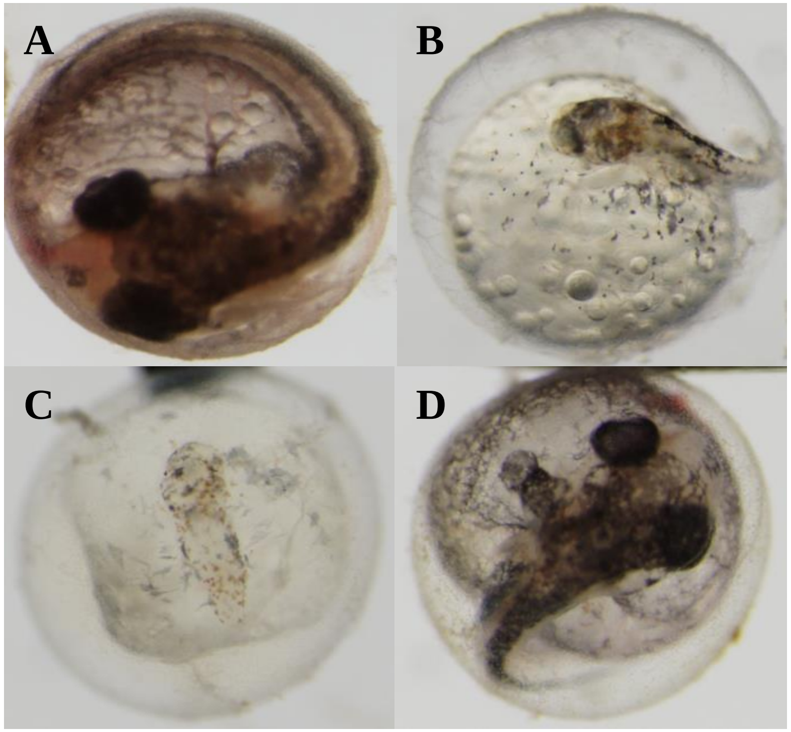

Figure S4. Unresolved gastrulation defect mutants (F_3 embryos 14 dpf). **A.** Wild-type. **B.** Delayed development and missing eyes/mouth/jaw phenotype (R149 family). **C.** Mass of non-patterned post-gastrula phenotype (R159 family). **D.** Lateral appendage phenotype (R240 family).
