# Supplementary material for: A Simultaneous Genetic Screen for Zygotic and Sterile Mutants in a Hermaphroditic Vertebrate (Kryptolebias marmoratus)
Source: G3 (Bethesda). 2016 Jan 20;6(4):1107–19. doi: 10.1534/g3.115.022475 (PMC4825645; doi:10.1534/g3.115.022475)
Supplement: Supporting Information [file supp_g3.115.022475_FigureS5.pdf]

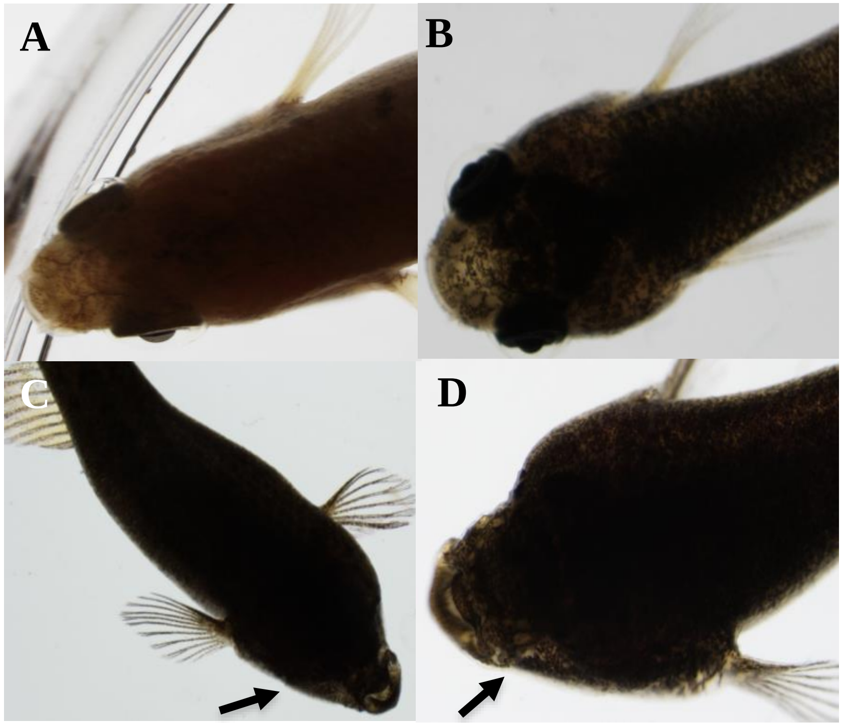

Figure S5. Hyperpigmentation mutants (mature adult fish). **A.** Wild-type. **B.** Hyperpigmented phenotype with normal eyes (R058 family). **C.** Hyperpigmented, eyeless and V-shaped jaw phenotype (R096 family). **D.** Hyperpigmented, eyeless and V-shaped jaw phenotype (R152 family). Arrows indicate the characteristic V-shaped jaw phenotype.
