# Supplementary material for: A Simultaneous Genetic Screen for Zygotic and Sterile Mutants in a Hermaphroditic Vertebrate (Kryptolebias marmoratus)
Source: G3 (Bethesda). 2016 Jan 20;6(4):1107–19. doi: 10.1534/g3.115.022475 (PMC4825645; doi:10.1534/g3.115.022475)
Supplement: Supporting Information [file supp_g3.115.022475_FileS1.pdf]

File S1

Fish Husbandry

K. marmoratus used for this study were maintained in the VSU Aquatic Laboratory in a controlled environment at 27-30º on a 14 h light:10 h dark photoperiod. Larvae and adult fish were housed in either 1-L plastic food containers or 2-L breeder tanks (Fisher Scientific, Pittsburgh, PA; LBTANK, Aquatic Ecosystems Inc., Apopka, FL) in 17-18 ppt Instant Ocean (Instant Ocean Inc., Earth City, MO) [17 g/L prepared using water treated by reverse osmosis (RO water)] with biweekly water changes to avoid algae accumulation. Air holes were added to 1-L lids and all holes were covered with 10 cm x 10 cm Black UV Stabilized 1/8" mesh with a glue stick to prevent escape of fish (Industrial Netting, Minneapolis, MN). Embryos were collected from the 2-L breeder tanks by first removing the adult hermaphroditic parents using the basket inserts followed by retrieval with 3-mL disposable polyethylene pipettes. Embryos were observed with an Olympus SZX16 stereomicroscope (Olympus, Center Valley, PA). They were kept in sterile 100 X 15 mm polyestyrene petri dishes (Fisherbrand, Pittsburgh, PA) containing brackish water until hatching and then transferred to ~ 200 mL of brackishwater in plastic food containers (Fisher Scientific, Pittsburgh, PA). Young hatchlings were reared in 250-mL containers for a month (to about 20 mm in standard length). Individuals were fed 1 mL of brine shrimp daily, with frequent water changes and subsequently transferred to either 1-L plastic containers for further growth and storage, or breeder tanks for embryo collection. Fish in 2-L breeder tanks were fed 3 mL of brine shrimp solution and fish in 1-L plastic containers were fed 2 mL, both every other day. Young hatchlings in 250-mL plastic containers were fed 1 mL daily. Fish were fed freshly hatched *Artemia nauplii*

(Artemac; Biomarine, Inc, Hawthorne, CA) harvested from a commercial grade hatchery funnel daily (BS6; Aquatic Ecosystems Inc., Apopka, FL). Shrimp were hatched in a 26-32 ppt solution of brackish water that was prepared dissolving 160.0 g of Instant Ocean in 5 L of RO water plus 10 g of cysts. Salinity was confirmed using a hydrometer and the hatchery funnel was provided with constant aeration. Cysts were given 24 h at constant aeration to hatch. In order to prepare them for feeding, the air hose was removed to stop aeration and the shrimp were allowed to settle for 10 min. Once they accumulated at the bottom, they were drained through a sieve made using 150 μm mesh to remove cyst shells (Aquatic Ecosystems Inc., Apopka, FL). Afterwards, brine shrimp were rinsed with zebrafish water (60 mg/1L Instant Ocean), and resuspended in 1.25 L of zebrafish water (~300 shrimp nauplii/mL). Unhatched cysts were left to settle to the bottom and discarded. This process was repeated twice. If an excess of unhatched cysts was still visible, they were removed with a 3-mL disposable plastic pipette.
